# Early prediction of end-stage kidney disease using electronic health record data: a machine learning approach with a 2-year horizon

**DOI:** 10.1093/jamiaopen/ooae015

**Published:** 2024-02-27

**Authors:** Panayiotis Petousis, James M Wilson, Alex V Gelvezon, Shafiul Alam, Ankur Jain, Laura Prichard, David A Elashoff, Naveen Raja, Alex A T Bui

**Affiliations:** UCLA Health Clinical and Translational Science Institute, David Geffen School of Medicine, University of California Los Angeles (UCLA), Los Angeles, CA 90024-2943, United States; Department of Medicine, David Geffen School of Medicine, University of California Los Angeles (UCLA), Los Angeles, CA 90024-2943, United States; UCLA Health Office of Health Informatics and Analytics, David Geffen School of Medicine, University of California Los Angeles (UCLA), Los Angeles, CA 90024-2943, United States; UCLA Health Office of Health Informatics and Analytics, David Geffen School of Medicine, University of California Los Angeles (UCLA), Los Angeles, CA 90024-2943, United States; UCLA Health Office of Health Informatics and Analytics, David Geffen School of Medicine, University of California Los Angeles (UCLA), Los Angeles, CA 90024-2943, United States; UCLA Health Office of Health Informatics and Analytics, David Geffen School of Medicine, University of California Los Angeles (UCLA), Los Angeles, CA 90024-2943, United States; Biostatistics and Computational Medicine, University of California Los Angeles (UCLA), Los Angeles, CA 90024-2943, United States; UCLA Health Faculty Practice Group and the Department of Medicine, David Geffen School of Medicine at University of California Los Angeles (UCLA), Los Angeles, CA 90024-2943, United States; Medical & Imaging Informatics (MII) Group, Department of Radiological Sciences, David Geffen School of Medicine, University of California Los Angeles (UCLA), Los Angeles, CA 90024-2943, United States

**Keywords:** machine learning deployment, early prediction ESKD model, electronic health record, end-stage kidney disease (ESKD)

## Abstract

**Objectives:**

In the United States, end-stage kidney disease (ESKD) is responsible for high mortality and significant healthcare costs, with the number of cases sharply increasing in the past 2 decades. In this study, we aimed to reduce these impacts by developing an ESKD model for predicting its occurrence in a 2-year period.

**Materials and Methods:**

We developed a machine learning (ML) pipeline to test different models for the prediction of ESKD. The electronic health record was used to capture several kidney disease-related variables. Various imputation methods, feature selection, and sampling approaches were tested. We compared the performance of multiple ML models using area under the ROC curve (AUCROC), area under the Precision-Recall curve (PR-AUC), and Brier scores for discrimination, precision, and calibration, respectively. Explainability methods were applied to the final model.

**Results:**

Our best model was a gradient-boosting machine with feature selection and imputation methods as additional components. The model exhibited an AUCROC of 0.97, a PR-AUC of 0.33, and a Brier score of 0.002 on a holdout test set. A chart review analysis by expert physicians indicated clinical utility.

**Discussion and Conclusion:**

An ESKD prediction model can identify individuals at risk for ESKD and has been successfully deployed within our health system.

## Introduction

End-stage kidney disease (ESKD) is a severe, permanent condition in which the kidneys no longer function. ESKD has significant healthcare and economic implications. In the United States, the number of individuals with ESKD increased by 41.8% between 2000 and 2019,^1^ with an estimated 785 000 people presently affected. This increase is largely due to an aging population and higher rates of diabetes, hypertension, and chronic kidney disease (CKD).^2^ Treatment and management of ESKD patients require dialysis or kidney transplant, representing a significant healthcare expenditure.^3^ Indeed, according to the United States Renal Data System (USRDS), 7% of all Medicare claims are due to ESKD.^1^ In 2021, expenditures from outpatient dialysis costs totaled 10 billion dollars.^4^ This condition has a significant impact on survival. Patients with ESKD live 25-30 years less than their healthy counterparts. Notably, there are also significant disparities in the prevalence and treatment of ESKD between racial and ethnic minority groups, with Black and Hispanic patients being affected the most.^5^

Closely monitoring the progression of CKD to ESKD is thus critical both to improve patients’ health and quality of life and to reduce the growing costs associated with this disabling disease. Effective management of hypertension and diabetes through medication or lifestyle changes can help prevent ESKD. Furthermore, the advent of sodium-glucose transport protein 2 inhibitors can reduce the risks of cardiovascular disease and slow the progression of CKD.^6^ Still, it is often unclear who is at high risk for rapid progression to ESKD, and identifying such individuals must occur early enough to give healthcare providers a chance to meaningfully intervene. Finding this group of high-risk patients is deemed crucial in curtailing the impact of ESKD in the United States.^1^

In this work, we present a novel data-driven approach for identifying and monitoring high-risk CKD patients for rapid progression to ESKD. We validated and conducted a comparison of our machine learning (ML) model with expert physicians through extensive chart review and compared our model with the well-established Kidney Failure Risk Equation (KFRE). The contributions of our work are 3-fold: (1) we use the entire medical history of an individual to generate a 2-year horizon prediction of ESKD; (2) we apply our model in a monthly moving window in the clinic, updating risk predictions across our health system population; and (3) we employ explainability methods to provide insights around a patient’s ESKD risk classification. We briefly describe how we translated our work into the clinic and discuss key points on predicting ESKD and monitoring our ML model in practice.

## Background

Tangri et al^7^^,^^8^ pioneered the development of 4- and 8-variable Cox proportional hazard regressions for the progression of CKD to kidney failure, called KFREs. KFRE-4 uses age, sex, estimated Glomerular Filtration rate (eGFR), and urine albumin-to-creatinine ratio (UACR); while KFRE-8 adds serum albumin, phosphate, bicarbonate, and calcium. External validation of the KFRE-4 equation by Whitlock et al^9^ highlights the issue of missing values for specific clinical variables (eg, UACR) for Stages 3-5 CKD patients and the lack of pre-existing conditions (eg, glomerulonephritis) as predictive variables by the KFREs. With these labs being ordered less frequently in CKD patients at Stages 1 or 2, missingness is typically higher, making it challenging to predict ESKD risk for low-stage CKD patients. However, earlier identification of high risk for progression provides an opportunity for more timely intervention. Table 1 summarizes various studies on the prediction of ESKD and their comparison with the KFREs. In summary, the use of ML improves the prediction of ESKD. Attempts by Bai et al^10^ and Chuah et al^11^ to build models from electronic health record (EHR) data demonstrated the utility of clinical variables. Bai et al^10^ used a longitudinal CKD cohort to highlight the importance of incorporating observations over time for ESKD prediction. Segal et al^12^ demonstrated that a model from claims data that includes pre-existing conditions can predict ESKD accurately. Nevertheless, Obermeyer et al^13^ emphasize that using healthcare cost as an input variable introduces race bias. Thus, a model combining clinical and claims data (ie, diagnoses, procedures, and medications) may be ideal. In this work, we used insights from these studies and developed the richest dataset to our knowledge to study the early prognosis of ESKD, incorporating observations over time to predict risk.

**Table 1.** Summary of literature review of ESKD predictive modeling studies.

Study	Results	Advantages	Disadvantages
Tangri et al^7^ 3449 patients (386 with kidney failure, 11%)	C-statistic at 2 years: KFRE-4, 0.91; KFRE-8, 0.92	High discrimination for ESKD prediction Simplicity	Overestimation of risk in some non-North American regions; calibration required for some areas CKD Stages 3-5 are population-specific
Tangri et al^8^ 721 357 participants, 23 829 cases of kidney failure	C-statistic KFRE-4: 0.90 over 2 years; 88 over 5 years	Externally validated in 30 countries spanning 4 continents	Potential specificity issues at certain risk thresholds
Whitlock et al^9^^,^^10^ 1512 included patients, 151 developed kidney failure	At 3% risk threshold: sensitivity, 97%; specificity: 62%. At 10% risk threshold: sensitivity, 86%; specificity, 80%	Performed well on external population	Lower specificity at lower risk thresholds High missingness (only 11.7% of Stage 3-5 CKD patients had UACR measurements) KFRE does not account for pre-existing conditions
Bai et al^10^ 748 CKD patients, ESKD was observed in 70 patients (9.4%)	AUCROC: logistic regression, 0.79; naïve Bayes, 0.80; random forest, 0.81; k-nearest neighbors, 0.73; decision tree, 0.66; KFRE-4, 0.80	Machine learning models had equivalent predictability with KFRE-4 in a CKD stage 1-5 cohort	Cohort of 1000 subjects Population CKD stage distribution does not reflect actual CKD population distribution Stage 1 CKD patients can result in a large number of false positives
Chuah et al^11^ ESKD cases (*n* = 263) and non-ESKD cases (*n* = 2125)	XGBoost vs KFRE-4: accuracy, 93.9% vs 91.3%; sensitivity, 60% vs 25%; specificity, 97.7% vs 97.6%; PPV, 75% vs 50%	Outperform KFRE-4 and KFRE-8 Use of SHAP values and feature importance Performance comparison of model and clinicians	21.5 years of EHR data used to select patients Focused on laboratory and vital features, ignoring pre-existing conditions
Segal et al^12^ 26 991 patients, ESKD was observed in 1585 patients (9.4%)	XGBoost: AUCROC, 0.93; PR AUC: 0.71	Effective use of medical insurance claims data for training and testing Large cohort of 550 000 patients CKD Stage1-4 patients Includes pre-existing conditions	Potential racial bias: use of cost as a predictive variable^13^ CKD and ESKD definitions are only through diagnosis codes Control group index date is the last available entry, which may result in many patients lost to follow-up

## Methods

Our ESKD model was developed from a dataset drawing on all outpatients seen as part of the University of California, Los Angeles (UCLA) Health System between 2013 and 2019. We excluded inpatient data to remove “sicker” individuals from consideration and the potential introduction of bias/dataset skew. The dataset, derived from our EHR, includes laboratory values, vitals, demographics, social history, International Classification of Disease (ICD) diagnoses, Current Procedural Terminology procedure codes, medication, and encounter data. Lab data include eGFR, serum and urine creatinine, calcium, urine protein creatinine ratio, blood and urine albumin, lipid panels, HbA1c, C3/C4 complements, erythrocyte sedimentation rate, C-reactive protein, blood and urine glucose, complete blood count panels, parathyroid hormone (PTH), vitamin D, electrolytes, blood urea nitrogen (BUN), and blood pH. Vitals include systolic and diastolic blood pressure, mean arterial pressure, pulse pressure, and weight. A history of specific conditions (Type 1/2 diabetes, hypertension, transplant, dialysis) was noted based on ICD codes and the status of transplant and renal dialysis. An expert panel drove the selection of the above variables. Tables S8 and S9 depict the population’s characteristics and important laboratory variables statistics.

### Data preprocessing

**Figure 1.**
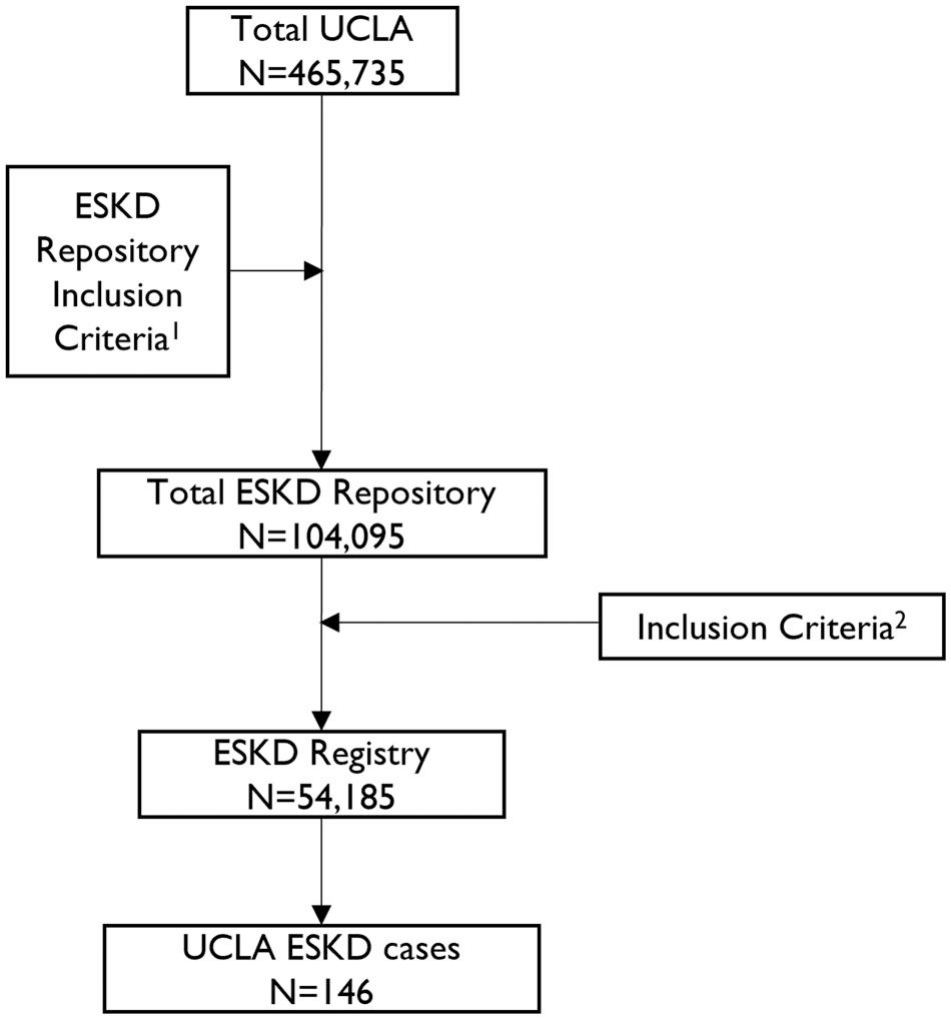
ESKD dataset Strobe diagram. Inclusion criteria 1: represents steps 1 and 2 in the Data preprocessing section. Inclusion criteria 2: represents steps 2-5. Abbreviation: ESKD, end-stage kidney disease.

Figure 1 depicts the STROBE diagram of our cohort. To operationalize a predictive model, our clinical experts first defined a CKD pattern as 2 consecutive eGFR readings below 90 at least 3 months apart. An ESKD outcome was then defined as any of the following (whichever occurred first) in the 2 years immediately after a CKD pattern: (1) any decrease in eGFR below 15 mL/min/1.73 m^2^; (2) a kidney transplant; and/or (3) initiation of dialysis. Patients with an eGFR below 15 but an acute kidney injury (AKI) occurring in the same period as an ESKD event in these 2 years were considered non-ESKD. Here, AKI was defined as any case whose eGFR recovered in 2 months. Only patients with 1 year of available EHR data before the CKD pattern were included in our analysis. We note that our definition of CKD differs from the Kidney Disease Improving Global Outcomes (KDIGO) definition of 2 consecutive eGFR readings below 60 at least 3 months apart (CKD pattern). Instead, ours was modified to an eGFR of 90 to include Stage II CKD patients in our predictive task. We excluded: (1) patients who died in the 2-year prediction period before being able to determine an outcome. There were 24 such patients that we excluded from the final dataset. A total of 21 patients out of 54 185 died after ESKD, and 877 out of 54 185 without ESKD died within 2 years. This is 0.04% and 1.62% of the whole population, respectively. (2) Any patient with ESKD before the CKD; (3) patients not affiliated with UCLA, either from a primary care physician (PCP) or nephrologist the year before the CKD pattern (ie, a record of at least one encounter with a UCLA-affiliated PCP or nephrologist); (4) patients younger than 18 and older than 100 years old; and (5) patients with ESKD in the same month as the CKD pattern. Applying the inclusion/exclusion criteria resulted in a total of 54 185 patients, with the proportion of ESKD cases calculated to be 0.27%. Of 54 185 patients, 53 515 (98.8%) had a follow-up encounter 1.5 years after CKD detection.

We aimed to create the best model for the early prediction of ESKD using 12 months of EHR data before the detection of CKD. Labs, vitals, patient visits, and social history data were preprocessed to reflect the monthly activity of each patient in each group. For example, for variables such as blood pressure or serum creatinine (continuous variables), the monthly average, minimum, maximum, standard deviation, and measurement count were computed. Additional feature engineering of variables includes the monthly one-over creatinine value, the CKD stage on the date of the detected CKD pattern (ie, date of second eGFR below 90), and the monthly difference between average, maximum, minimum, standard deviation, and measurement counts over time. ICD codes for the year before the CKD pattern were grouped into Clinical Classification Software Refined (CCSR) disease categories using the Health Cost and Utilization Project (HCUP) classification system,^14–18^ resulting in 498 disease categories. Additionally, 231 chronic disease category groups were created. Procedure codes were also grouped into 213 Clinical Classification Software (CCS) categories. Medications for the year before the CKD pattern were grouped into the Epic Systems pharmaceutical subclasses, resulting in 643 medication categories. Each diagnosis, procedure, and medication category represents the frequency of occurrence of a diagnosis, procedure, and medication for each patient. Drug refills, doses, and dispenses were also computed monthly for the 12 months before the CKD pattern. Serum creatinine was used to estimate the number of AKIs monthly in the year before the CKD pattern according to the 2012 KDIGO criteria. Data preprocessing resulted in 6229 features.

EHR data suffer from missing data based on various missingness mechanisms.^19^ To deal with missing values in temporal labs or vitals, we used linear interpolation from the initial month till the end of the 12 months.^20–22^ When the initial value of a lab or vital was missing, we initiated the linear interpolation method with the population’s average. Simple mean imputation was used to impute the missing values of other static variables.^23^^,^^24^ Min-max scaling was used to scale all features between [0,1].

### Model development and evaluation

**Figure 2.**
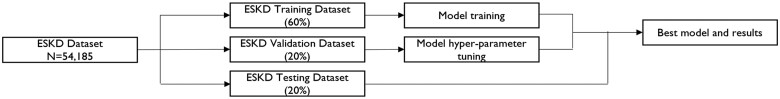
Evaluation and tuning approach.

We randomly split our dataset into training (60%), validation (20%), and holdout test sets (20%) stratified in terms of the outcome (ESKD event) and CKD stage at prediction time (Figure 2). Multiple strategies were used to assess and compare different ML methods. Our approach to selecting the best ESKD model involved comparing linear and nonlinear models, models trained on datasets with low proportion of missing values, and models trained on datasets with imputed values.

*A parsimonious logistic regression model*: This model used only variables with <5% missing values. This model included CKD pattern eGFR values, eGFR slope, and demographic variables.

*Models generated based on feature selection*: Feature selection was performed on the training data using the Pearson correlation coefficient and user-selected thresholds to filter out less correlated variables. Correlation thresholds in this list of values [0.1, 0.075, 0.05, 0.005, 0.0] (ie, iteratively, features with a correlation coefficient smaller than each threshold were dropped from the dataset) were used to select the most relevant features. Logistic regression (LR), random forest (RF), and gradient-boosting machine (GBM) models were compared. A grid search and a randomized grid search were applied to find the best LR, and RF and GBM models, respectively. Randomized grid search was used^25^ to deal with the large number of combinations of hyperparameters. Additionally, an LR model based on the top 8 most important clinical features of the GBM model was assessed (features include the latest reading of serum creatinine, eGFR, urine protein creatinine ratio (PCR), CKD stage, UACR, PTH, number of AKIs in the past month, and BUN). All model details are provided in Table S6 and Figure S8.

During model training, under-, over-, and no-sampling of the minority class were evaluated. Model tuning details are outlined in Table S5. For comparison, we also evaluated the KFRE-4 equation^9^ on the test set. The model development and evaluation pipelines were built using Python 3.7 and the scickit-learn^26^ and XGBoost^27^ ML libraries for model development.

### Model selection

The validation and test set evaluation results were used to select the best model and report model performance on the holdout test set, respectively. The best model selection was performed on the validation dataset using the PR-AUC metric, which is appropriate for imbalanced datasets as it highlights the model’s precision in identifying the rare events of ESKD.

## Results

**Figure 3.**
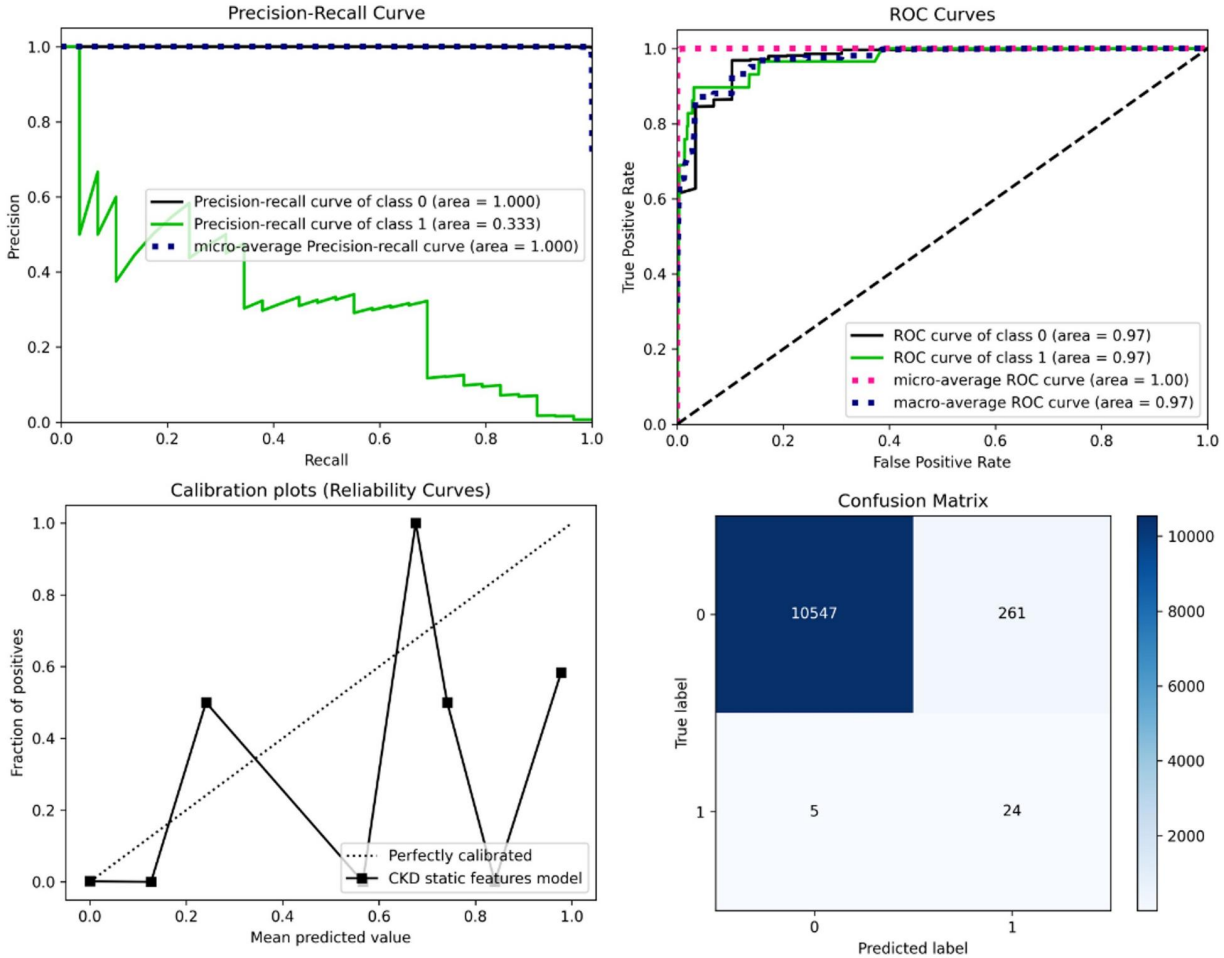
Model performance curves and confusion matrix on the test set. Class 0: Non-ESKD cases. Class 1: ESKD cases. Abbreviation: ESKD, end-stage kidney disease.

**Figure 4.**
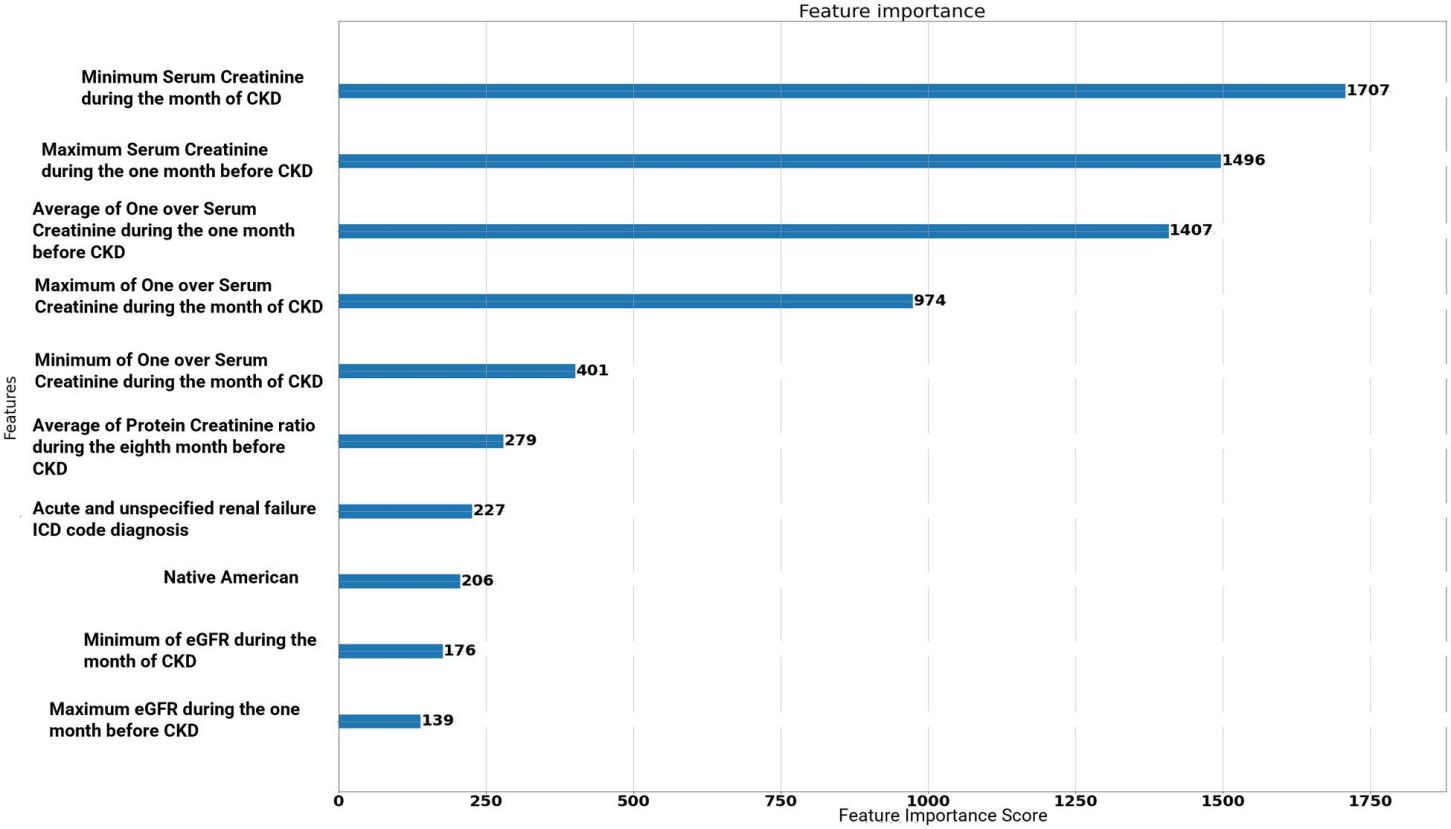
Top 10 most important features based on information gain.

Table 2 shows the PR-AUC on the validation set for each model. Table S10 shows all models’ evaluation metrics results using the test set. The best model is a GBM model using the XGBoost library with a PR-AUC of 0.568 on the validation set. Table 3 depicts additional performance metrics for this model using an optimal probability threshold of 0.001688 on the hold-out test set. This threshold was selected using the validation set to maximize true positives and minimize false positives. The model had an area under the ROC curve (AUCROC) of 0.971 on the hold-out test set, suggesting excellent discrimination. Figure 3 depicts the ROC, PR, and calibration curves on the test set. This model was trained on a randomly oversampled training set with 1:1 ESKD to non-ESKD events. Subsequently, using a correlation threshold of 0.1, we selected a subset of 335 features out of the 6229 to train the model. Model training was optimized on PR-AUC and log-likelihood. Figure S6 depicts model convergence on both metrics. Early stopping was employed to avoid overfitting, with the model converging optimally on both the training and validation datasets. Furthermore, the results depicted in Tables 2 and 3 are after model calibration. Model feature importance is shown in Figure 4. Clinical features at the point of prediction are most important when predicting ESKD. Figure S7 shows the geometric decrease in feature importance. Regardless, at the individual level, feature importance varies. To this end, we used the Shapley permutation explainer^28^ to generate individualized explanations of feature importance on each model prediction. This method is model agnostic, computing Shapley values for any model by iterating over complete permutations of the features forward and backward (ie, features with high Shapley values are interpreted as driving the individualized model prediction).

**Table 2.** PR-AUC metrics of best models based on the validation and test set.

		Feature selection		PR-AUC	
Model type	Sampling method	Correlation threshold	Train	Validation	Test
LR parsimonious	Under-sampling	—	0.289	0.242	0.274
LR	Over-sampling	0.1	0.423	0.502	0.403
RF	Over-sampling	0.0	1.00	0.563	0.178
LR using GBM 8 most important features	Over-sampling	—	0.469	0.464	0.351
KFRE 4 var equation	—	—	0.417	0.338	0.293
GBM	**Over-sampling**	**0.1**	**1.00**	**0.568**	**0.333**

The validation set is used to select the best model. Bold: best performing model.

**Table 3.** GBM model’s additional performance metrics using the optimal threshold of 0.001688 on the test set.

Brier score	F1 score	PR-AUC	Precision/PPV	ROC-AUC	Recall/sensitivity	Specificity
0.002	0.153	0.333	0.084	0.971	0.828	0.976

### Error analysis and expert chart review

We optimized our model on the PR-AUC metric during training to enhance model precision when detecting rare outpatient ESKD cases. Given this imbalance, our calibration curve is unstable, and our PR-AUC indicates that a prediction of an ESKD case will result in additional false positive cases. This finding motivated the need for a chart review, as well as an assessment for clinical utility.

We performed a chart review of patients from the test set with an expert nephrologist. The expert was blinded to the outcome and was allowed to use the entire EHR before the prediction point to classify a patient as ESKD or not. After the expert’s prediction, the expert generated an updated “ground truth” that includes “clinical positive” cases. Clinical positives are patients who do not follow our predefined rule of an ESKD case but still need close monitoring. Chart review was performed randomized on a total sample of 82 patients. The sample was randomly picked around the model’s threshold selection point to include difficult non-ESKD cases. Table 4 shows the model performance against the expert nephrologist. Interestingly, most false positives were classified by our expert nephrologist as “clinical positives” patients, with their kidney filtration rate significantly close to becoming ESKD, thus needing close monitoring. Moreover, a significant portion of false positive ESKD patients have ESKD in a period longer than 2 years (eg, at 25, 26, 36, or 48 months).

**Table 4.** Chart review results: top: expert performance; bottom: model performance.

Expert vs ground truth					
*N* = 82	Precision (PPV)	Recall (sensitivity)	F1-score	Number of samples	Accuracy
ESKD/Clinical positive	1.00	0.71	0.83	49	0.83
Non-ESKD	0.70	1.00	0.82	33	

**GBM model vs ground truth**					
ESKD/Clinical positive	0.90	0.96	0.93	49	0.91
Non-ESKD	0.93	0.85	0.89	33	

### Fairness/bias analysis

A fairness analysis was performed on race/ethnicity, sex assigned at birth, age, and social vulnerability index (SVI). In Table S7, we outline the proportion of cases for each category of the variable of interest over the confusion matrix, the ground truth, and the model’s average probability. Subsequently, we used the Pearson chi-square test to assess the independence of the categories between groups. For instance, we assessed independence between the race distribution of ESKD and non-ESKD cases. A *P*-value <.05 supports the rejection of the null hypothesis of independence. Race, age, and SVI were not statistically independent with respect to the event of ESKD. Interestingly, given our model’s average probability per category of each variable, the model also captures race, age, and SVI differences, ensuring non-biased and fair predictions.

## Discussion

When CKD progresses to its final stage of ESKD, treatment options are limited, and individuals face a reduced quality of life and increased risk of comorbidities, including ischemic heart disease, congestive heart failure, malnutrition, poor functional status, and increased risk of mortality.^29^ Moreover, the financial cost of treatment is significant and continues to grow: at our academic medical center, ESKD is amongst the highest-costing medical conditions. However, predicting the onset of ESKD is notoriously difficult, despite the past decade of effort in this area. While it is important to provide additional support to all patients with CKD, the reality of resource constraints in healthcare requires identifying those at high risk so that care management resources can be allocated accordingly and slowing CKD progression. We addressed this issue through a successful collaboration between biomedical data scientists and informaticians, implementation scientists, physicians, nurses, social workers, care coordinators, and operations within our health system to create a pragmatic, data-driven solution. Engaging stakeholders and experts, we collectively defined the problem, identified meaningful predictions that could be clinically actionable, and operationalized a validated model to provide proactive guidance.

**Figure 5.**
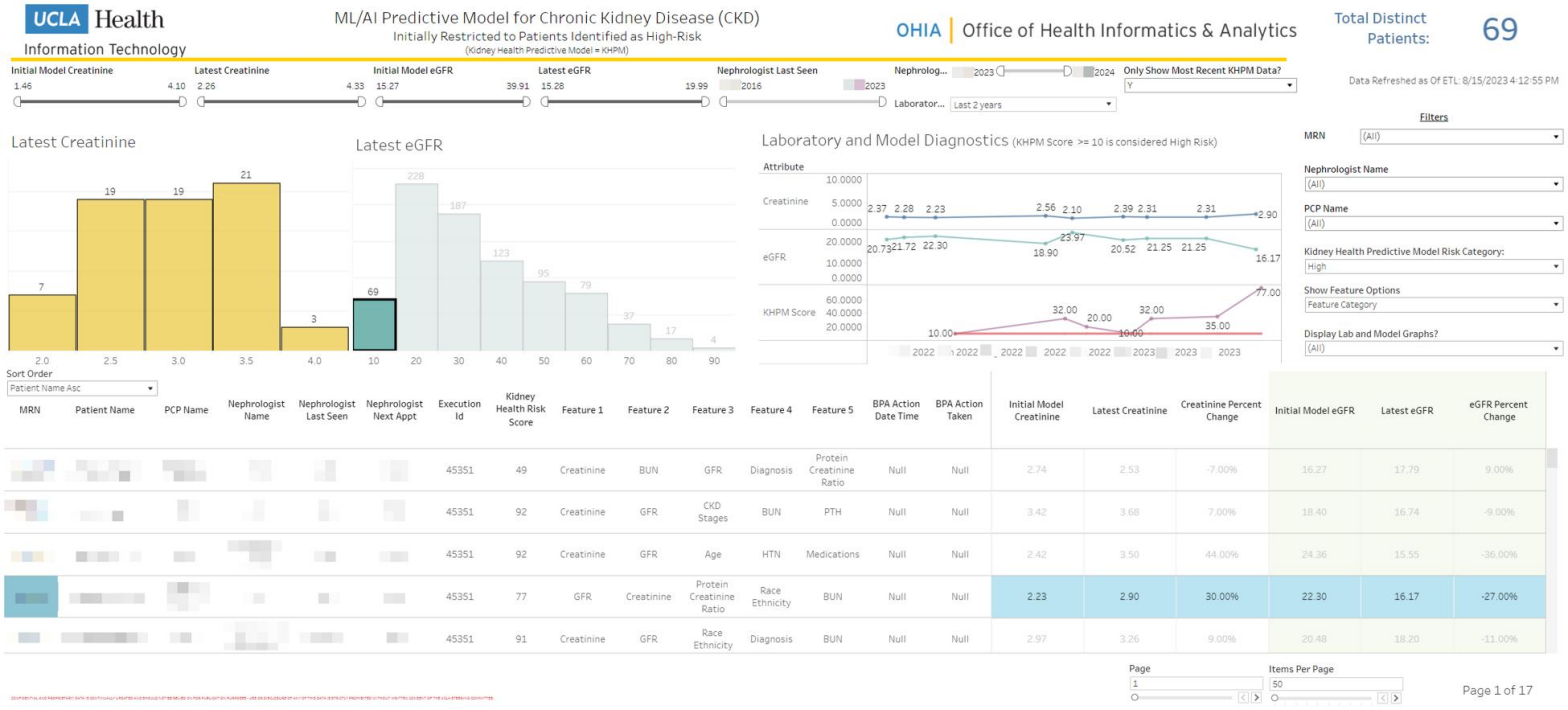
Dashboard screenshot used in the high care ESKD clinic. Left distribution of creatinine and eGFR of predicted ESKD patients by the model. Laboratory and model diagnostics represent the time points the model started to follow a high-risk ESKD patient over time after they have been flagged as ESKD with a risk score ≥10. The red line represents a risk score threshold of 10. Risk score is the normalized model probability from 0 to 100. The purple line represents the time points this patient was tested by the model. The green line represents recorded eGFR measurements, and the blue line represents the equivalent serum creatinine measurements. Table columns feature 1 to feature 5 represent individually tailored explainability results of the 5 most influential variables for the model’s Kidney Health Risk Score. Abbreviation: ESKD, end-stage kidney disease.

The final product is a 2-year horizon ESKD disease model that uses the EHR history of an individual as input. Notably, our best model uses both clinical variables associated with ESKD and those captured over time, achieving a high AUCROC of 0.97, a PR-AUC of 0.33, and high calibration. The model was compared in a blind chart reviewed with our expert nephrologist’s prediction on a set of the most difficult ESKD and non-ESKD cases to discriminate, with the model outperforming the expert predictions in several cases. Overall, the model is more accurate with slightly lower precision. A combination of a group of expert nephrologists and the model will significantly improve and optimize the care of ESKD patients. This model is now deployed in our institution’s health system and EHR as an information support tool. The model is run monthly for every patient that fulfills the CKD pattern (ie, 2 consecutive eGFR readings below 90 at least 3 months apart). Patients deemed high risk are automatically flagged for review by UCLA’s Division of Nephrology. A dashboard was developed to facilitate review (Figure 5). Individualized explanations of the model’s prediction are provided to the patient’s assigned nephrologist. Additionally, as monthly predictions are accrued, an ESKD risk profile of a patient is created, depicting the trend of risk overtime on a medical dashboard that is part of the EHR. Nephrologists receive notifications of the risk of individual patients among their patient population via the EHRs messaging system (In Basket).

The inclusion criteria for our cohort were based on patient affiliation with our institution for a year, outpatient status, and CKD Stages 2-4. This definition resulted in having a large pool of patients with a disproportionately small group of ESKD patients, as most ESKD patients are inpatient. Including Stage 2 CKD patients makes the model cohort significantly imbalanced. Nevertheless, reflecting real-world scenarios when training a model is critical for its effectiveness in the clinic. Our goal was to train a model that performs best in early prognosis using all available EHR data. The KFRE in Shpaner et al^30^ tends to improve performance in patients with progressed CKD. Similarly, LR, RF, and a parsimonious LR model performed comparably to our best model. Still, given the small number of ESKD patients, we must identify a model with the highest, most stable performance. Interestingly, when building an LR model from the top 8 most important clinical features identified from our best model, its performance was comparable to the best model, and we look to externally validate it in future work. The best model we developed is an institution-specific application that uses all available kidney-related features to improve early ESKD prediction, making it practical and well-suited to routine local clinical practice with individualized explanations. This point stresses the growing dichotomy between the use of data-driven solutions, including ML-based models, to be used to address local operations vs more generalizable knowledge. In this case, we opt for the former to optimize care for our patients.

The development of ML models for clinical support, particularly when using EHR-based data, faces numerous challenges:

- Standardizing the dataset and dealing with missing values is problematic when dealing with observational clinical data. Such issues can occur when different tests are ordered by myriad providers across institutions when patients miss visits, or follow-ups are ordered at irregular time intervals, etc. Moreover, different missing data mechanisms are observed in the EHR, such as missing completely at random, missing at random (MAR), or missing not at random (MNAR).^19^^,^^20^ Here, we dealt with time-series (eg, labs and vitals) missing data as MNAR and in cases of missing static data (eg, sex, race) as MAR. We used linear interpolation and mean imputation to deal with missing data. Our parsimonious LR model uses minimal imputation and has a PR-AUC of 0.242 on the validation set compared to the rest of the models that employ imputation as part of their pipeline; they all have a PR-AUC above 0.3 in the validation set. Hence, imputation improves model performance in this scenario, likely by capturing patient variance.
- The cohort of patients for our analysis has a prevalence of ESKD patients of 0.27%. This low number of ESKD cases complicated model development. To avoid overfitting, we had to split our dataset into a stratified training, validation, and test set. The stratification was based on the outcome of ESKD *and* the CKD stage of the patient to maintain a consistent distribution of severe patients across each set. To boost training performance, we used feature selection and over-sampling. The AUCROC indicates that our model can discriminate against non-ESKD patients very accurately, hence reducing the number of false positives to something manageable by the capacity of our health system.
- The CRISP-DM framework was used for model development and deployment.^31^ When developing our model, ethical considerations were important. Individualized explanations were necessary both from a clinical and a patient perspective. As such, this was a motivating factor for the development of a rich (feature-wise) dataset to support SHAP value individualized explanations. Furthermore, we performed a comprehensive fairness/bias analysis to ensure unbiased and transparent model predictions.

Applying a model in the clinic requires rigorous testing. Our model predictions are continuously monitored and compared against physicians’ interventions. In this work, we employed a chart review with expert nephrologists, recording their decisions on the outcome of difficult cases around our model’s decision threshold to justify model tuning.

Future work will require developing methods that enable a learning health system with faster integration and feedback on the model’s performance and use of different data sources. Moreover, there is a need to improve the current explainability techniques in healthcare, fostering an understanding of model predictions and furthering trust. The model was officially deployed in the summer of 2023, with data continuously collected around model drift and patient outcomes. Within the context of this ESKD model, we are conducting prospective data collection comparing the nephrologists’ evaluation versus the model predictions and plan to perform a 2-year complete evaluation comparing any changes in model performance from the development cohort and the impact of the model on local healthcare operations. This task is complicated as the EHR continuously changes; new procedures and interventions are added, new definitions of specific labs are introduced, and the number of observations increases.^32^

We recognize that our approach has several limitations. First, we focused on an outpatient population, which inherently is a highly imbalanced classification/detection problem. However, this population presents the most opportunity to slow progression toward ESKD. This low event rate may further introduce challenges in generalizing the model to other institutions or populations. Recognizing this, part of our future work will be applying our LR model based on the top 8 most important features to datasets from other sites, identifying how differences in environments may affect model performance.

Still, given the increasing rate of ESKD nationally, any reduction in that rate would be desirable and would showcase the effectiveness of the model to help prevent ESKD events.

## Supplementary Material

ooae015_Supplementary_Data

## Data Availability

The data underlying this article will be shared on reasonable request to the corresponding author.
