# Supplementary material for: Early prediction of end-stage kidney disease using electronic health record data: a machine learning approach with a 2-year horizon
Source: JAMIA Open. 2024 Feb 27;7(1):ooae015. doi: 10.1093/jamiaopen/ooae015 (PMC10898824; doi:10.1093/jamiaopen/ooae015)
Supplement: ooae015_Supplementary_Data [file ooae015_supplementary_data.docx]

# SUPPLEMENTARY MATERIAL


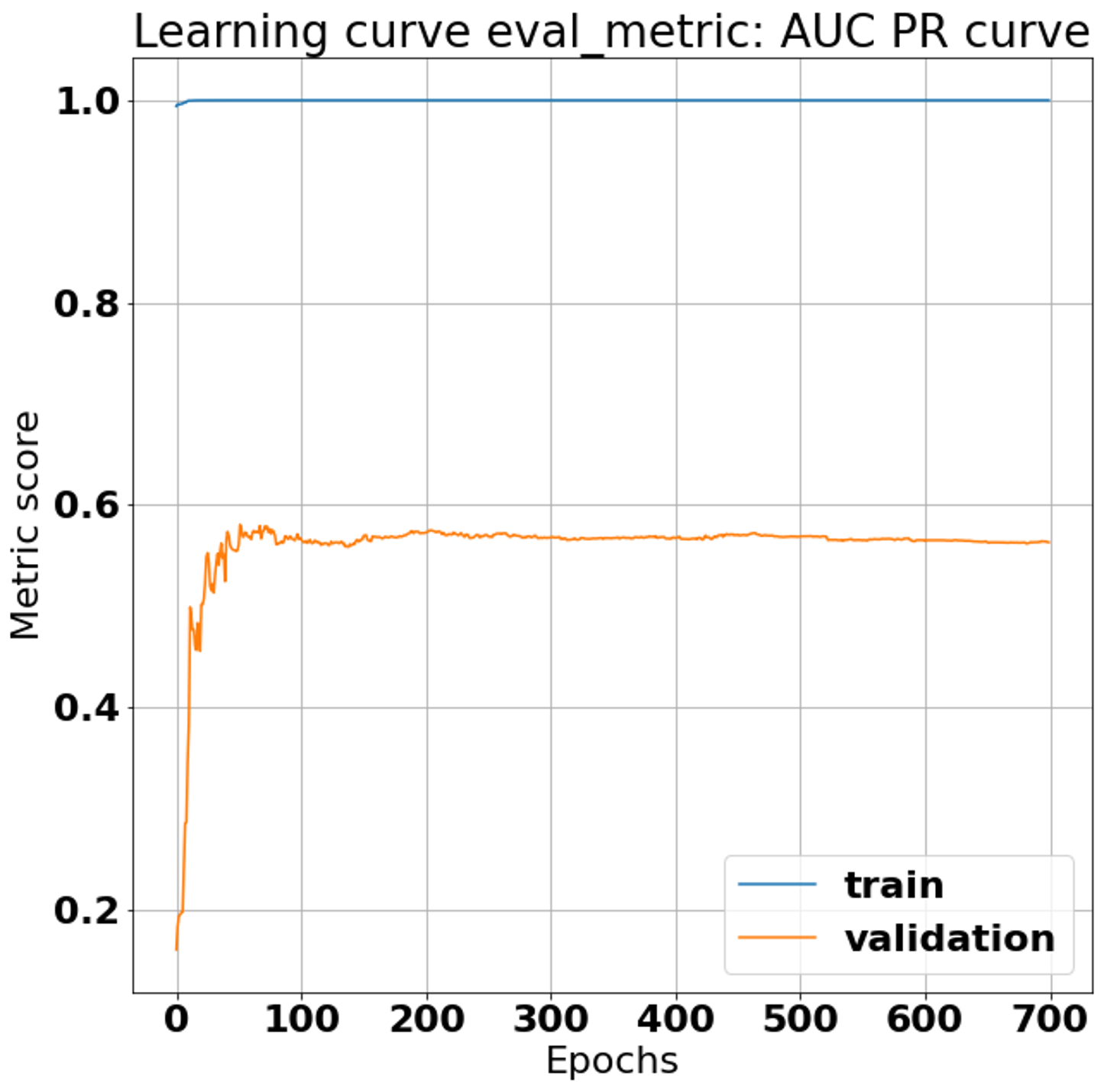

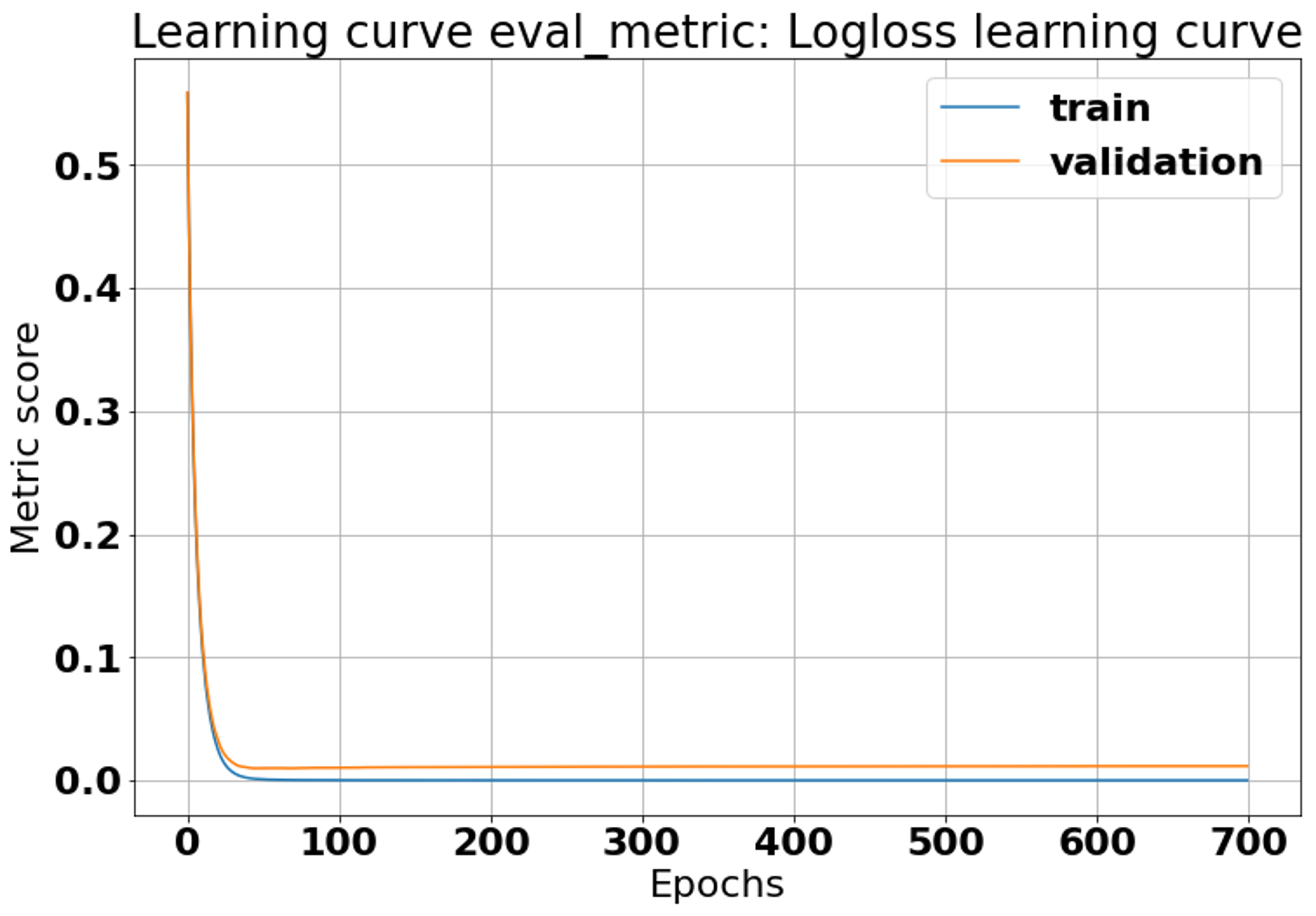


Figure 6 Model optimal fit on the training and validation set.


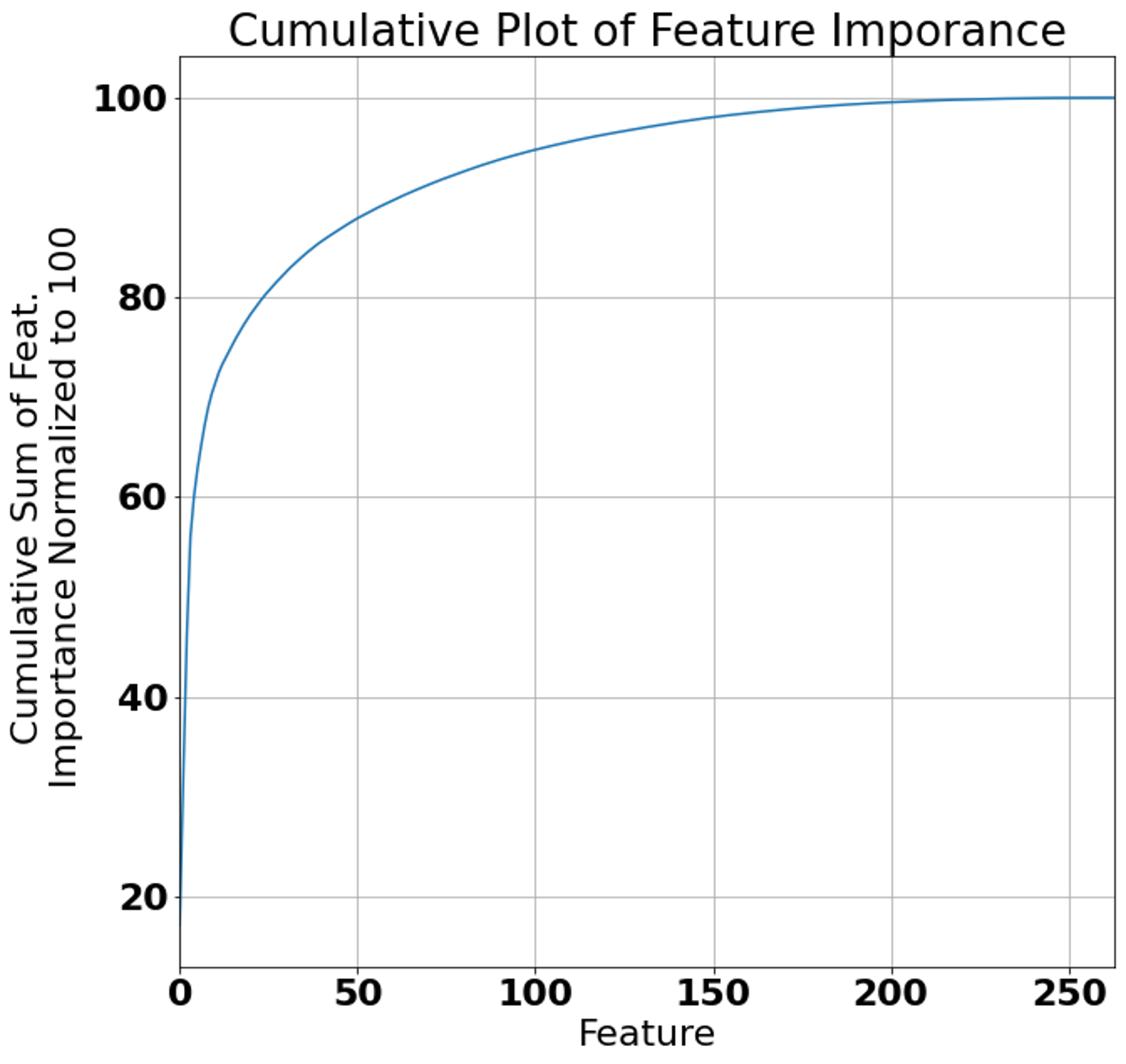


Figure 7 Cumulative plot of feature importance. Most important feature at 0.

Table 5 Model type hyperparameter space.

| Type of model | Hyperparameter space |
| --- | --- |
| Logistic Regression | C: logspace(-4, 1) in 5 steps |
| Random Forest | bootstrap: [True, False],max_depth: [10, 30, 100],max_features: ["auto", "sqrt"],min_samples_leaf: [1, 2],min_samples_split: [2, 5],n_estimators: (10, 100) in increments of 10, and (100, 1050) in increments of 100,criterion: ["gini", "entropy"] |
| Gradient Boosting Machine | learning_rate: [0.001, 0.05, 0.10, 0.15],max_depth: [8, 10, 12],min_child_weight: [1],gamma: [0.5, 0.8, 1],colsample_bytree: [0.5, 0.7, 0.9],early_stopping_rounds: [10],reg_alpha: [0.001, 0.05, 0.10, 0.15],reg_lambda: [0.001, 0.05, 0.10, 0.15],n_estimators: (10, 100) in increments of 10 and(100, 1550) in increments of 100 and(2000, 10050) in increments of 1000,} |

Table 6 LR model based on the top eight most important clinical features of the GBM model. Scale feature values before using weights/coefficients using (X – min)/(max-min).

| **Features within the month prior to prediction** | **Weights/coefficients** | **Max** | **Min** |
| --- | --- | --- | --- |
| **CKD stage (1-5)** | 0.50584132 | 5 | 2 |
| **Sr. Creatinine / mg/dL** | 0.23160628 | 4.8 | 0.5 |
| **Number of AKIs** | 0.07649979 | 5 | 0 |
| **UPCR** | 0.03810137 | 16876.5 | 0 |
| **PTH / pg/mL** | 0.03509055 | 770 | 2 |
| **UACR** | 0.02360416 | 12143 | 1 |
| **BUN / mg/dL** | 0.01717749 | 858 | 2 |
| **eGFR / mL/min/1.73m^2^** | -0.31592322 | 108.005 | 15.523 |


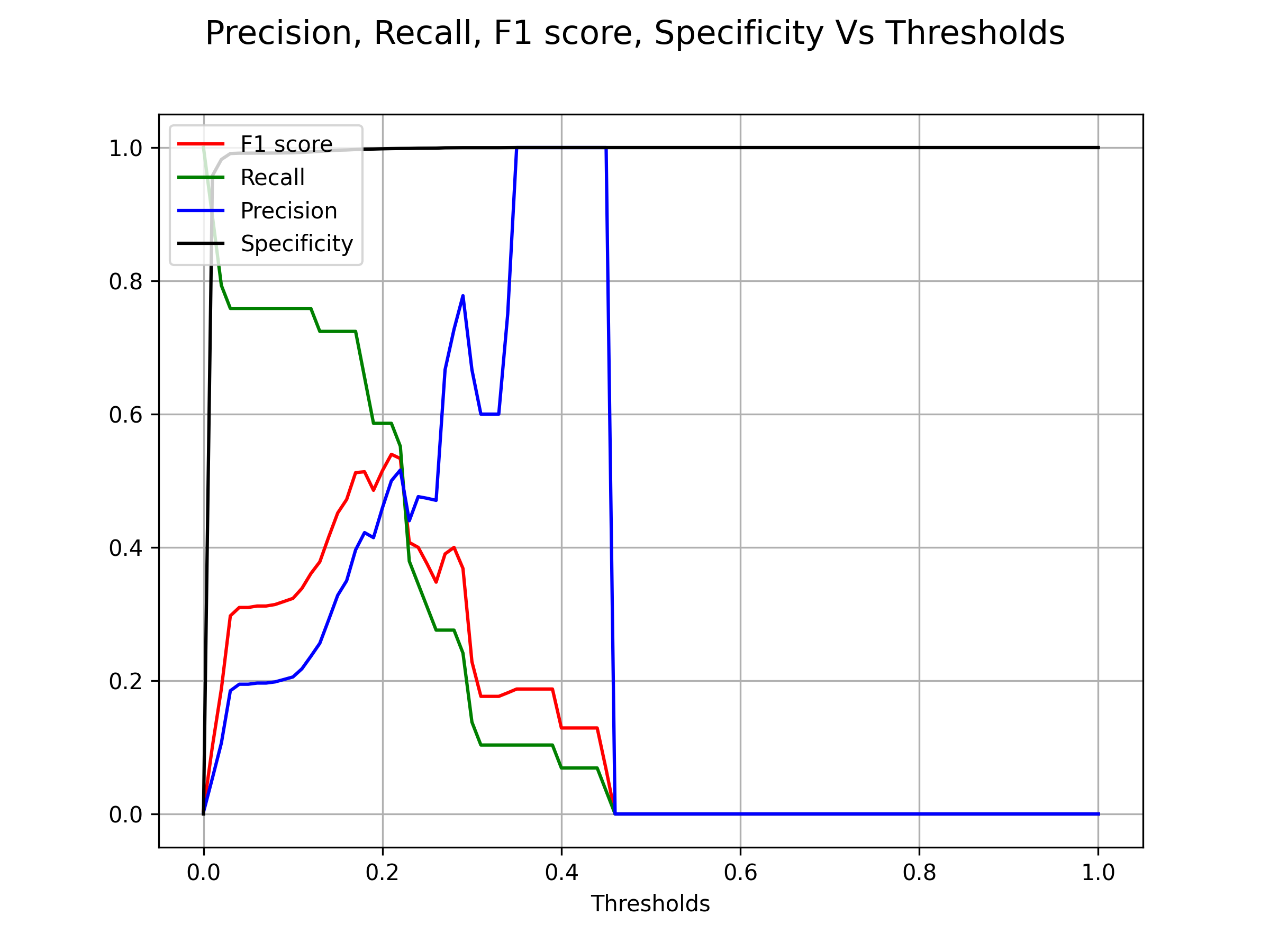


Figure 8 LR model based on the top eight most important clinical features of the GBM model Precision, Recall, F1 score, and Specificity plot. Based on a hospital’s resources, a threshold can be used to classify individuals as experiencing an ESKD event in the following two years. A probability threshold of 0.2 results in a similar recall, precision, F1 score, and high specificity. A threshold lower than 0.2 results in high recall, and a higher than 0.2 in higher precision.

Table 7 Fairness/Analysis. The chi-square test was performed to assess variable independence with respect to the outcome and independence between the confusion matrix groups. A p-value < 0.05 indicates that the null hypothesis of independence is rejected.

|  | Confusion Matrix Percentages | | | | Ground Truth Percentages | | Model average probability per group | | |
| --- | --- | --- | --- | --- | --- | --- | --- | --- | --- |
| Race / Ethnicity (p < 0.05) | **FNs** | **FPs** | **TNs** | **TPs** | **ESKD** | **NON-ESKD** | **Total - population** | **ESKD** | **NON-ESKD** |
| Asian | 0.00 | 9.91 | 4.03 | 10.14 | 9.59 | 4.16 | 0.0050 | 0.4893 | 0.0020 |
| Black | 0.00 | 12.53 | 5.32 | 13.04 | 12.33 | 5.48 | 0.0057 | 0.4805 | 0.0028 |
| Islanders | 0.00 | 0.25 | 0.17 | 0.72 | 0.68 | 0.17 | 0.0122 | 0.5756 | 0.0063 |
| Native | 0.00 | 0.76 | 0.19 | 1.45 | 1.37 | 0.20 | 0.0121 | 0.5758 | 0.0017 |
| Other race | 37.50 | 27.52 | 41.14 | 26.09 | 26.71 | 40.84 | 0.0024 | 0.3758 | 0.0018 |
| White | 62.50 | 49.03 | 49.15 | 48.55 | 49.32 | 49.15 | 0.0031 | 0.4349 | 0.0019 |
| Hispanic | 12.50 | 12.28 | 8.33 | 20.29 | 19.86 | 8.42 | 0.0049 | 0.4185 | 0.0023 |
| Multiracial | 12.50 | 3.64 | 5.15 | 2.17 | 2.74 | 0.51 | 0.0022 | 0.2888 | 0.0017 |
| Sex Assigned at Birth (p > 0.05) |  |  |  |  |  |  |  |  |  |
| Male | 100 | 47.65 | 46.86 | 44.44 | 46.15 | 46.87 | 0.0028 | 0.48886 | 0.00185 |
| Female | 0 | 52.35 | 53.11 | 55.56 | 53.85 | 53.10 | 0.0026 | 0.415882 | 0.001806 |
| Unknown | 0 | 0 | 0.03 | 0 | 0.00 | 0.03 | 0.0017 |  | 0.001686 |
| Choose not to disclose | 0 | 0 | 0.00 | 0 | 0.00 | 0.00 | 0.0017 |  | 0.001686 |
| Age (p < 0.05) |  |  |  |  |  |  |  |  |  |
| 18 - 30 | 12.50 | 3.21 | 1.50 | 5.07 | 5.48 | 1.54 | 0.0070 | 0.404 | 0.003 |
| 30 - 40 | 0 | 4.48 | 5.52 | 7.25 | 6.85 | 5.50 | 0.0035 | 0.443 | 0.002 |
| 40 - 50 | 12.50 | 6.94 | 12.81 | 21.74 | 21.23 | 12.69 | 0.0043 | 0.494 | 0.002 |
| 50 - 60 | 0 | 11.76 | 25.06 | 13.77 | 13.01 | 24.77 | 0.0025 | 0.504 | 0.002 |
| 60 - 70 | 37.50 | 18.36 | 26.89 | 13.77 | 15.07 | 26.70 | 0.0024 | 0.365 | 0.002 |
| 70 - 80 | 25.00 | 19.20 | 17.84 | 17.39 | 17.81 | 17.87 | 0.0028 | 0.399 | 0.002 |
| 80 - 90 | 12.50 | 23.60 | 8.61 | 16.67 | 16.44 | 8.94 | 0.0040 | 0.391 | 0.002 |
| 90 - 100 | 0.00 | 12.44 | 1.76 | 4.35 | 4.11 | 1.99 | 0.0043 | 0.477 | 0.002 |
| SVI Rank (p < 0.05) |  |  |  |  |  |  |  |  |  |
| 0 - 10 | 25.00 | 18.05 | 26.46 | 16.54 | 17.02 | 26.28 | 0.0025 | 0.401 | 0.002 |
| 10' - 20 | 37.50 | 17.07 | 19.26 | 12.03 | 13.48 | 19.21 | 0.0027 | 0.452 | 0.002 |
| 20 - 30 | 0.00 | 16.71 | 16.32 | 18.05 | 17.02 | 16.33 | 0.0029 | 0.366 | 0.002 |
| 30 - 40 | 0.00 | 10.28 | 11.47 | 10.53 | 9.93 | 11.45 | 0.0028 | 0.453 | 0.002 |
| 40 - 50 | 25.00 | 10.81 | 8.14 | 6.77 | 7.80 | 8.20 | 0.0031 | 0.469 | 0.002 |
| 50 - 60 | 0.00 | 5.81 | 5.28 | 5.26 | 4.96 | 5.29 | 0.0031 | 0.454 | 0.002 |
| 60 - 70 | 0.00 | 5.72 | 4.40 | 7.52 | 7.09 | 4.43 | 0.0049 | 0.569 | 0.002 |
| 70 - 80 | 0.00 | 6.43 | 3.81 | 9.02 | 8.51 | 3.87 | 0.0050 | 0.409 | 0.003 |
| 80 - 90 | 12.50 | 5.81 | 3.26 | 12.03 | 12.06 | 3.32 | 0.0068 | 0.430 | 0.003 |
| 90 - 100 | 0.00 | 3.31 | 1.59 | 2.26 | 2.13 | 1.63 | 0.0038 | 0.576 | 0.002 |

Table 8 Cohort characteristics. Race, ethnicity, sex, and CKD stage are reported as percentages, and age, eGFR, and serum creatinine are reported with the mean and standard deviation. The missing values column represents the percentage of missing values per variable.

| Race - % |  | Missing values - % |
| --- | --- | --- |
| White | 49.14 | 0 |
| Asian | 4.17 | 0 |
| Black | 5.5 | 0 |
| Islanders | 0.18 | 0 |
| Native | 0.2 | 0 |
| Other races | 40.8 | 0 |
| Multiracial - % | 5.11 | 0 |
| Ethnicity - % |  | 0 |
| Hispanic | 8.45 |  |
| Sex - % |  | 0 |
| Female | 53.21 |  |
| Male | 46.79 |  |
| CKD stages - % |  |  |
| 2 | 85.89 | 0 |
| 3 | 9.58 | 0 |
| 4 | 3.48 | 0 |
| 5 | 1.04 | 0 |
| Age – mean (std) | 61.2 (14.3) | 0 |
| CKD pattern 1st eGFR – mean (std) | 74.73 (13.11) | 0 |
| CKD pattern 2nd eGFR – mean (std) | 74.08 (13.48) | 0 |
| Sr. Creatinine (of CKD pattern 2nd eGFR)-– mean (std) | 1.03 (0.26) | 0 |

Table 9 The mean and standard deviation of other important clinical characteristics of the cohort. Statistics before and after imputation depict the change in variance after imputation. The missing values column represents the percentage of missing values per variable.

|  | Before Imputation | | After Imputation | | Missing values - % |
| --- | --- | --- | --- | --- | --- |
|  | **Mean** | **Standard**  **deviation** | **Mean** | **Standard**  **deviation** |  |
| UACR | 226.06 | 699.04 | 142.81 | 200.75 | 97.13 |
| UPCR | 750.31 | 1710.83 | 460.67 | 300.14 | 97.75 |
| PTH | 62.52 | 46.03 | 56.29 | 11.21 | 96.18 |
| BUN | 17.88 | 10.13 | 17.85 | 10.24 | 5.16 |
| Number of AKIs | 0.03 | 0.2 | 0.03 | 0.2 | 0 |

Table 10 Models evaluated. Performance metrics on hold-out test set.

| Model-type | Brier score loss | F1-score | PR-AUC | Precision-score | ROC-AUC | Recall-score | Specificity-score | Accuracy | TN | TP | FN | FP |
| --- | --- | --- | --- | --- | --- | --- | --- | --- | --- | --- | --- | --- |
| LR using GBM 8 most important features | 0.002 | 0.067 | 0.351 | 1.000 | 0.948 | 0.034 | 1.000 | 0.997 | 10808 | 1 | 28 | 0 |
| GBM | 0.002 | 0.153 | 0.333 | 0.084 | 0.971 | 0.828 | 0.976 | 0.975 | 10547 | 24 | 5 | 261 |
| KFRE 4 var equation | 0.002 | 0.005 | 0.293 | 0.003 | 0.950 | 1.000 | 0.000 | 0.003 | 0 | 29 | 0 | 10808 |
| LR parsimonious | 0.002 | 0.065 | 0.274 | 0.500 | 0.949 | 0.034 | 1.000 | 0.997 | 10807 | 1 | 28 | 1 |
| RF | 0.002 | 0.005 | 0.178 | 0.003 | 0.952 | 1.000 | 0.000 | 0.003 | 0 | 29 | 0 | 10808 |
| LR | 0.002 | 0.436 | 0.403 | 0.462 | 0.954 | 0.414 | 0.999 | 0.997 | 10794 | 12 | 17 | 14 |
